# D-Dimer in Acute Mesenteric Venous Thrombosis: A Prospective Case-Control International Multicenter Study

**DOI:** 10.1177/11772719241296631

**Published:** 2024-11-26

**Authors:** Stefan Acosta, Annika Reintam Blaser, Alexandre Nuzzo, Yasmin Soltanzadeh-Naderi, Joel Starkopf, Alastair Forbes, Marko Murruste, Kadri Tamme, Anna-Liisa Voomets, Merli Koitmäe, Miklosh Bala, Zsolt Bodnar, Dumitru Casian, Zaza Demetrashvili, Alan Biloslavo, Virginia Dúran Muñoz-Cruzado, Benjamin Hess, Karri Kase, Mikhail Kirov, Matthias Lindner, Cecilia I Loudet, Dimitrios Damaskos, Martin Björck, AMESI Investigators (Collaborators), Olivier Corcos

**Affiliations:** 1 Department of Clinical Sciences, Malmö, Lund University, Sweden; 2 Institute of Clinical Medicine, University of Tartu, Estonia; 3 Department of Intensive Care Medicine, Lucerne Cantonal Hospital, Switzerland; 4 Intestinal Stroke Center, Department of Gastroenterology, IBD and Intestinal Failure, AP-HP. Nord, Beaujon Hospital, Paris Cité University, Paris, France; 5 Tartu University Hospital, Estonia; 6 Estonian Genome Center, Institute of Genomics, University of Tartu, Estonia; 7 Institute of Mathematics and Statistics, University of Tartu, Estonia; 8 Hadassah Medical Center and Faculty of Medicine, Hebrew University of Jerusalem, Jerusalem, Israel; 9 Letterkenny University Hospital, Letterkenny, Ireland; 10 Nicolae Testemitanu State University of Medicine and Pharmacy of the Republic of Moldova, Chisinau, Moldova; 11 N. Kipshidze Central University Hospital, Tbilisi, Georgia; 12 University Hospital of Trieste ASUGI, Trieste, Italy; 13 Virgen del Rocío University Hospital, Sevilla, Spain; 14 Northern State Medical University and City Hospital #1, Arkhangelsk, Russia; 15 Universitätsklinikum Schleswig-Holstein, Campus Kiel, Kiel, Germany; 16 Hospital General San Martin de La Plata, Buenos Aires, Argentina; 17 Royal Infirmary of Edinburgh, Edinburgh, UK; 18 Department of Surgical Sciences, Vascular surgery, Uppsala University, Uppsala, Sweden

**Keywords:** Mesenteric venous thrombosis, diagnosis, suspected acute mesenteric ischaemia, D-dimer

## Abstract

**Background:**

Acute mesenteric venous thrombosis (MVT) is rarely suspected as primary diagnosis in emergency departments and still carries an in-hospital mortality rate of above 20%.

**Objectives:**

The aim of this study was to find differences in clinical and laboratory markers between patients with acute MVT and a control group of suspected but confirmed as not having any type of acute mesenteric ischaemia (AMI).

**Design:**

Data was retrieved from the AMESI (Acute MESenteric Ischaemia) study. This international, multicenter prospective case-control study from 32 sites collected data on patients with suspected AMI during a 10-month period.

**Methods:**

Independent factors associated with acute MVT were evaluated in a multivariable logistic regression analysis and expressed as odds ratios (OR) with 95% confidence intervals (CI).

**Results:**

D-dimer was not significantly higher in MVT (n = 73) compared to non-AMI (n = 287) patients (median 7.0 mg/L vs 4.5 mg/L, *P* = .092). After entering BMI, atherosclerotic disease, history of venous thromboembolism, CRP, and D-dimer as covariates in a multi-variable logistic regression analysis, absence of atherosclerotic disease (OR 0.096, 95% CI 0.011-0.84; *P* = .034) and elevated D-dimer (OR 2.59/one SD increment, 95% CI 1.07-6.28; *P* = .034) were associated with MVT. The discriminative ability of D-dimer for MVT as assessed by area under the curve in the receiver operating characteristics analysis was 0.63 (95% CI 0.49-0.78).

**Conclusion:**

Elevated D-dimer was associated with MVT, but the discriminative ability of D-dimer was poor. There is an urgent need to find a more accurate plasma biomarker for this condition.

**TRIAL REGISTRATION:**

NCT05218863 (registered 19.01.2022).

## Plain Language Summary

Acute mesenteric vein thrombosis is rare, but life-threatening. The symptoms are abdominal pain and are not distinct from other acute abdominal conditions. A blood test identifying or leading to easier diagnosis is warranted. This multicenter international study has included patients with thrombosis in the mesenteric veins as well as suspected diseases, but not confirmed conditions, of disturbed circulation to the gastrointestinal tract. Elevation of the blood marker D-dimer was found to be associated with the condition of acute mesenteric vein thrombosis, but was not enough discriminative in relation to those patients with suspected disease only. There is an urgent need to find a more accurate blood marker for deep vein thrombosis of the abdomen.

## Introduction

Acute mesenteric venous thrombosis (MVT) is very rarely^1^ suspected as diagnosis based on clinical and laboratory data in emergency departments, and MVT still carries an in-hospital mortality of above 20% in some reports.^2^ Studies on diagnostic performance of plasma biomarkers in acute MVT have not been available since studies on biomarkers often include all patients with acute mesenteric ischaemia (AMI), where most patients have acute superior mesenteric artery (SMA) occlusion.^3^ Patients with acute MVT versus SMA occlusion have very different clinical presentations, risk factor profiles, disease severity and prognosis,^4^ and it is therefore not appropriate to pool data on these 2 disease entities in clinical research.

The AMESI (Acute MESenteric Ischaemia) study is an international prospective multicentre trial.^5^ This study includes patients with all subtypes of AMI as well as suspected but confirmed as non-AMI. The incidence, management, and outcome of AMI in the AMESI study has recently been outlined.^6^

The aim of this study was to perform a prospective case-control study to evaluate clinical data and laboratory markers of MVT among patients with suspected AMI, for potential facilitation of patient selection for definitive diagnosis with computed tomography (CT) with intravenous contrast phase enhancement and imaging in the portal phase.

## Methods

### Study design

**Figure 1.**
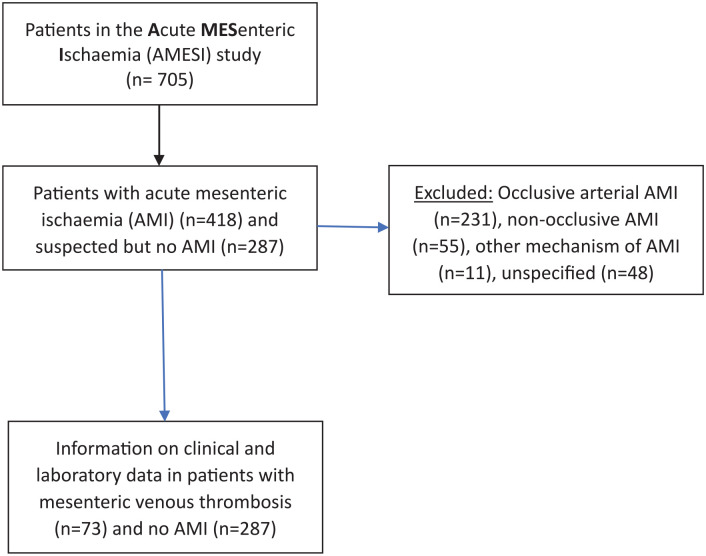
Flow chart of included study patients.

The study design has been outlined in detail.^6^ The AMESI study is an international, multicenter, prospective study and included patients with all subtypes of AMI as well as suspected AMI eventually confirmed as non-AMI. If suspicion of AMI was not confirmed or mechanical bowel obstruction with local intestinal gangrene was the final diagnosis, only baseline data and hospital mortality was collected. The present sub-study is a post-hoc analysis of the AMESI study (Figure 1).

### Study sample

In brief, patients over 18 years of age with suspected or confirmed AMI were either admitted or transferred to 32 hospitals between 06-06-2022 and 05-04-2023. This sub-study complied with the Strengthening the Reporting of Observational studies in Epidemiology (STROBE) statement for cohort studies (Supplemental Table S1).

### Study objectives

To compare clinical and laboratory data between patients with confirmed MVT and control group of patients with suspected but confirmed not to have any subtype of AMI.

### D-Dimer assays

The test principle, manufacturer, name of D-dimer assay and reference values for each respective site providing data for this sub-study is outlined in Table 1.

**Table 1.** D-dimer assays in study sites including patients with acute mesenteric venous thrombosis.

Site Nr	Site name	Country	Test principle	Manufacturer	Name of D-dimer assay	Reference values
1	Tartu Ülikooli Kliinikum	Estonia	Automated immunoassay (immunoturbidometry)	Diagnostica Stago	STA Liatest^®^ D-Di Plus	<0.5 mg/L
2	Põhja-Eesti Regionaalhaigla	Estonia	Automated immunoassay (immunoturbidometry)	Diagnostica Stago	STA Liatest^®^ D-Di Plus	<0.5 mg/L
3	City Hospital, Arkhangelsk,	Russia	Automated immunoassay (immunoturbidometry)	Siemens	Innovance^®^ D-dimer	<0.45 mg/L
6	School of Medical Sciences & Hospital, University Sains Malaysia	Malaysia	Automated immunoassay (immunoturbidometry)	Diagnostica Stago	STA Liatest^®^ D-Di Plus	<0.45 mg/L
7	Hospital Melaka	Malaysia	Site investigators did not reply			
10	Luzerner Cantonsspital	Switzerland	Automated immunoassay (immunoturbidimetry)	Werfen	HemosIL^®^ D-dimer HS 500	<0.5 mg/L FEU
11	Hadassah Medical Center and Faculty of Medicine, Hebrew University of Jerusalem	Israel	Automated immunoassay (immunoturbidimetry)	Siemens	Innovance^®^ D-Dimer	<0.45mg/L
12	Universitätsklinikum Schleswig-Holstein, Campus Kiel	Germany	Automated immunoassay (immunoturbidimetry)	Werfen	HemosIL^®^ D-dimer HS 500	<0.5 mg/L
15	Virgen del Roc’o University Hospital, Seville	Spain	Automated immunoassay (immunoturbidimetry)	Werfen	HemosIL^®^ D-Dimer HS 500	<0.5 mg/L
17	Letterkenny University Hospital	Ireland	Automated immunoassay (immunoturbidimetry)	Siemens	Innovance^®^ D-Dimer	<0.5 mg/L
18	Erciyes University Hospital	Turkey	Automated immunoassay (immunoturbidimetry)	Siemens	Innovance^®^ D-Dimer	<0.5 mg/L
19	State University of Medicine and Pharmacy “Nicolae Testemitanu”	Moldova	Automated immunoassay Flourescence immunoassay	Boditech Med Inc (South Korea)	AFIAS D-Dimer test	⩽0.5 mg/L
24	Fujian Hospital	China	Automated immunoassay	Long Island Biotech, Shanghai, (China)	Mindray ExC810	⩽0.55 mg/L
31	Azienda Ospedaliera Universitaria Careggi, Florence	Italy	Site investigators did not reply			
34	Azienda Ospedaliero Universitaria Citta della Salute e della Scienza, Turin	Italy	Automated immunoassay (immunoturbidimetry)	Werfen	HemosIL^®^ D-dimer HS 500	<0.580 mg/l FEU
36	Royal Infirmary of Edinburgh	Scotland, United Kingdom	Automated immunoassay (immunoturbidimetry)	Siemens	Innovance^®^ D-Dimer	⩽0.25 mg/L
37	Beaujon Hospital, Paris	France	Automated immunoassay (immunoturbidimetry)	Diagnostica Stago	STA-Liatest^®^ D-Di plus	<0.5 mg/L

Abbreviation: FEU, fibrinogen equivalent units.

### Definitions

Suspicion of AMI was raised by the local investigators in an adult patient with acute abdominal pain, or critically ill patients in the intensive care unit with increasing plasma lactate levels and suspicion of non-occlusive mesenteric ischaemia (NOMI). Confirmation of AMI was assured by CT scan, endoscopy, surgery, histology or autopsy. Patients with mechanical bowel obstruction comprised collection of baseline data and hospital survival. Disability was categorized as need of assistance (no/yes). Atherosclerotic disease was defined as previous ischaemic heart disease, stroke and/or peripheral arterial disease (carotid artery disease, lower extremity arterial disease). Charlson co-morbidity index^7^ was calculated (mdcalc.com/calc/3917/charlson-comorbidity-index-cci). Bowel emptying^8^ was defined as diarrhoea and/or vomiting.

#### Statistics

Categorical data was presented as number and proportions (%) and continuous data as medians with interquartile ranges (IQR). Variables associated with MVT compared to non-AMI patients in univariable analysis (*P* < .1), were candidates for inclusion in the multi-variable logistic regression analysis, expressed in odds ratios (ORs) with 95% confidence intervals (CI). Normality of data was assessed by the Kolmogorov-Smirnov test, and all tested continuous variables were log10 transformed due to skewed distribution, and converted to *Z* scores, before entering as covariates into a multivariable logistic regression model. Continuous data after multi-variable testing were expressed per one standard deviation (SD) increment. A maximum of one covariate per ten MVT events was allowed in the multi-variable model. The clinical prediction multivariable model included pre-specified candidate predictors based on clinical experience. No imputation of missing data was done. The number of positive and negative D-dimer tests, according to the respective manufacturers normal reference range, in the MVT and non-AMI group, were analysed in a 2 × 2 contingency table, yielding sensitivity and specificity of D-dimer for MVT with 95% CI. Receiver operating Characteristics (ROC) analysis was performed to obtain an area under the curve (AUC) as a measurement of D-dimer, white blood cell counts and CRPs ability to discriminate between MVT and non-AMI. Level of statistical significance was *P* < .05. IBM SPSS Statistics, version 28 (SPSS, Chicago, IL, USA) was used for statistical analysis.

## Results

### Comparison of baseline demographics and risk factors between patients with acute MVT and control group

The total number of patients with confirmed acute MVT was 73, and the total number of patients with suspected but confirmed as non-AMI was 287, of which 128 suffered mechanical bowel obstruction. Compared to the control group, patients with MVT were younger (*P* = .008), had higher BMI (*P* = .009), lower Charlson comorbidity index (*P* = .003), less hypertension (*P* < .001), less atherosclerotic disease (*P* < .001), less atrial fibrillation (*P* = .006), received less antiplatelet drugs (*P* = .014), and had more previous venous thromboembolic event (*P* < .001; Table 2).

**Table 2.** Comparison of clinical background data between patients with acute MVT and confirmed non-AMI.

Patient characteristics, comorbidity and medical therapy	Acute MVT (n = 73)	Confirmed non-AMI (n = 287)	*P*-value
Age, years (median, IQR)	64 (48-74)	69 (56-80)	.008
Female gender (%)	28 (38.4)	138/284 (48.6)	.12
Body Mass Index, kg/m^2^ (median, IQR)	25.9 (23.4-31.5) (n = 56)	24.7 (22.0-27.7) (n = 216)	.009
Disability (%)	5/69 (7.2)	75/269 (27.9)	<.001
Current smoking (%)	17/66 (25.8)	51/225 (22.7)	.60
Hypertension (%)	27 (37.0)	169/280 (60.4)	<.001
Atherosclerotic disease (%)	9/72 (12.5)	96/269 (35.7)	<.001
Atrial fibrillation (%)	6 (8.2)	65 (22.6)	.006
Myocardial infarction (%)	3/69 (4.3)	33/274 (12.0)	.062
Previous venous thromboembolic event (%)	13/71 (18.3)	12/272 (4.4)	<.001
Charlson comorbidity index (median, IQR)	2 (1-4) (n = 64)	4 (2-5) (n = 268)	.003
Anticoagulant drugs (%)	20/72 (27.8)	56/270 (20.7)	.20
Antiplatelet drugs (%)	10/72 (13.9)	76/271 (28.0)	.014
Statins (%)	15/72 (20.8)	85/270 (31.5)	.078

### Comparison of clinical data at admission between acute MVTs and control group

The median pre-hospital symptom duration was 32 hours for patients with MVT compared to 24 hours for the control group (*P* = .040), and frequency of acute abdominal pain was higher in the MVT group (*P* = .042; Table 3).

**Table 3.** Comparison of clinical data at admission between patients with acute MVT and confirmed non-AMI.

Symptoms	Acute MVT (n = 73)	Confirmed non-AMI (n = 287)	*P-*value
Pre-hospital symptom duration, hours (median; IQR)	32 (13-72) (n = 56)	24 (6-48) (n = 190)	.040
Acute abdominal pain (%)	69 (94.5)	246 (85.7)	.042
Diarrhoea (%)	12 (16.4)	30 (10.5)	.16
Bloody stool (%) (macroscopic)	5 (6.8)	18 (6.3)	.86
Vomiting (%)	7 (9.6)	18 (6.3)	.32
Bowel emptying (%)	17 (23.3)	47 (16.4)	.17
Shock (%)	4 (5.5)	34 (11.8)	.11

Bowel emptying = diarrhoea and/or vomiting.

### Comparison of laboratory data between acute MVT and control group

**Figure 2.**
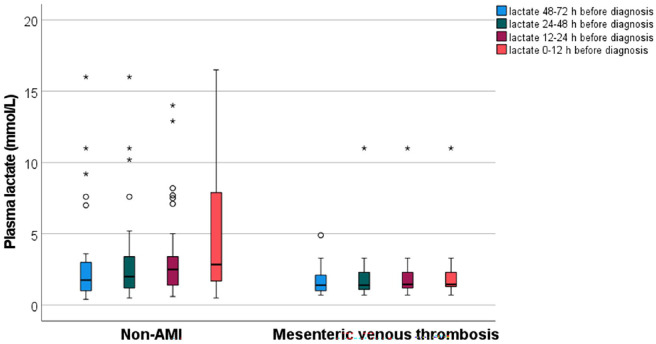
Box plot graph showing plasma lactate levels in time intervals prior to diagnosis. Temporal plasma lactate values prior to diagnosis of acute MVT versus non-AMI (acute mesenteric ischaemia) patients. Box plot graph showing median and interquartile range values of plasma lactate at different time ranges before diagnosis of acute MVT and non-AMI patients. The line across the box indicates the median, the box represents interquartile range, and the whiskers are lines that extend from the box edges to the highest and lowest values, excluding outliers. Values more than 1.5 IQR’s but less than 3 IQR’s (o), and more than 3 IQR’s (*), from the box edges are labelled as outliers and extremes, respectively

White blood cell count (*P* = .016) and C-reactive protein (CRP) level (*P* < .001) were more elevated in the acute MVT group compared to the control group. The AUC for white blood cell counts and CRP for MVT were 0.67 (95% CI 0.60-0.74) and 0.59 (95% CI 0.52-0.67), respectively. Estimated glomerular filtration rate (eGFR) at admission was higher in the MVT group (*P* < .001). Amylase (*P* = .033) and troponin T (*P* = .039) levels were lower in MVT patients. D-dimer was measured in 23 patients (31.5%) with MVT compared to 34 (11.8%) in the non-AMI group (*P* = .0001). D-dimer was not significantly higher in MVT compared to the control group (median 7.0 mg/L vs 4.5 mg/L, *P* = .092), whereas it was higher (*P* = .001) compared to patients with mechanical bowel obstruction (median 1.5 mg/L; n = 15). D-Dimer values were above the test’s respective reference value in all 23 tested patients with MVT, and in 27 out of 34 tested non-AMI patients, resulting in a sensitivity of 100% (95% CI 85.2-100) and specificity of 20.6% (95% CI 8.7-37.9). The discriminative ability of D-dimer for MVT as assessed by the AUC was 0.63 (95% CI 0.49-0.78). The optimum cut-off point for D-dimer performance of MVT diagnosis based on the ROC analysis was 5.0 mg/L. Plasma lactate levels at time point 12 to 24 hours before diagnosis (*P* = .007) and time point 0 to 12 hours before diagnosis (*P* = .005) were lower in MVT compared to the control group, and plasma lactate levels increased more between these 2 time-points in the control group (Table 4, Figure 2).

**Table 4.** Comparison of laboratory data between patients with acute MVT and confirmed non-AMI.

Laboratory data	Acute MVT (n = 73)	Confirmed non-AMI (n = 287)	*P*-value
White blood cell count, ×10^9^/L (median, IQR)	14.7 (10.6-21.9) (n = 70)	12.5 (8.0-16.8) (n = 277)	.016
CRP, mg/L (median, IQR)	105 (41-166) (n = 60)	44 (7.5-118.5) (n = 201)	<.001
eGFR, ml/min/1.73m^2^ (median, IQR)	82 (53-104) (n = 41)	60 (36-85) (n = 206)	<.001
ASAT, U/L (median, IQR)	28 (20-37) (n = 60)	28 (19-55) (n = 187)	.51
Amylase, U/L (median, IQR)	45 (27-55) (n = 34)	57 (34-122) (n = 140)	.033
Troponin T, ng/L (median, IQR)	12 (10-21) (n = 14)	38 (10-183) (n = 64)	.039
pH (median, IQR)	7.39 (7.32-7.45) (n = 47)	7.37 (7.27-7.42) (n = 209)	.090
Base excess, (median, IQR)	−1.4 (-4.3 to 1.4) (n = 28)	−1.8 (-8.0 to 1.2) (n = 203)	.33
D-dimer, mg/L (median, IQR)	7.0 (4.0-9.0) (n = 23)	4.5 (1.3-8.2) (n = 34)	.092
D-dimer above reference for normal value according to manufacturer (%)	23/23 (100)	27/34 (79.4)	.034
Time point 1. Lactate, mmol/L (median, IQR) (48-72 hours before diagnosis)	1.4 (1.0-2.1) (n = 28)	1.7 (1.3-3.0) (n = 37)	.10
Time point 2. Lactate, mmol/L (median, IQR) (24-48 hours before diagnosis)	1.4 (1.1-2.3) (n = 27)	2.2 (1.2-3.2) (n = 42)	.11
Time point 3. Lactate, mmol/L (median, IQR) (12-24 hours before diagnosis)	1.5 (1.2-2.3) (n = 34)	2.3 (1.4-3.7) (n = 75)	.007
Time point 4. Lactate, mmol/L (median, IQR) (0-12 hours before diagnosis)	1.6 (1.3-2.9) (n = 56)	2.4 (1.5-4.8) (n = 212)	.005
Repeated Lactate measurements (at least on two occasions) (%)	33/58 (56.9)	79/218 (36.2)	.004
Change in Lactate, mmol/L (median, IQR) between time point 4 and 1	0.0 (0.0-0.4) (n = 27)	0.50 (-0.22 to 2.15) (n = 34)	.062
Change in Lactate, mmol (median, IQR) between time point 4 and 3	0.0 (0.0-0.0) (n = 33)	0.30 (-0.05 to 1.60) (n = 73)	.008

### Clinical and laboratory variables associated with acute MVT

After entering body mass index, atherosclerotic disease, history of venous thromboembolism, CRP and D-dimer as covariates in a multi-variable logistic regression analysis, absence of previous atherosclerotic disease (OR 0.096, 95% CI 0.011-0.84; *P* = .034) and D-dimer (OR 2.59/one SD increment, 95% CI 1.07-6.28; *P* = .034) were associated with MVT (Table 5).

**Table 5.** Clinical and laboratory variables associated with acute MVT compared to confirmed non-AMI.

Variable	Multi-variable logistic regression	
	OR (95%CI)	*P*-value
Body mass index	2.42^a^ (0.87-6.76)	.090
Atherosclerotic disease	0.096 (0.011-0.84)	.034
History of venous thromboembolism	4.36 (0.20-93.50)	.35
C-reactive protein	1.65^a^ (0.41-6.57)	.48
D-dimer	2.59^a^ (1.07-6.28)	.034

All 5 variables were entered into the model.

a OR was expressed per one standard deviation increment.

## Discussion

This is the first ever prospective case-control study on patients with acute MVT. This is very difficult to achieve, due to the low incidence and awareness of the disease, preventing researchers from performing studies of higher quality, but was made possible thanks to data from the AMESI study. This sub-study on MVT patients, with a control group of patients, with similar symptoms but with proven non-AMI, has shown that elevated D-dimer and absence of previous atherosclerotic disease were independently associated with MVT in the adjusted analysis, which should alert clinicians to the possibility of MVT in a patient with acute abdomen. However, a clinical decision tool such as the Wells score^9^ for pre-test probability of deep vein thrombosis^10^ does not exist for MVT, and it is unlikely to ever be developed. Even if the Wells score cannot be used as a standalone test to confirm or exclude deep vein thrombosis, it is a valuable tool in combination with D-dimer measurement for evidence-based decision making.^11^ D-dimer had, however, a poor discriminative ability for diagnosis of MVT in the present study. This may be explained by the fact that only 23/73 patients with MVT had their D-dimer tested, and that the median time elapsed from debut of symptoms until presentation was 32 hours. D-dimer is an acute phase protein that will be more elevated close to the thrombotic event.

Although a history of atherosclerotic disease was independently inversely associated with MVT in the present study, this factor is of very little value in the clinical setting. There are, however, other factors not studied, such as known inherited thrombophilia, especially factor V Leiden mutation, that can give a clue towards the diagnosis of MVT. The prevalence of factor V Leiden mutation without presence of cancer was reported to be high in both MVT and systemic venous thromboembolism in a population-based study, suggesting that screening for thrombophilia should be considered in unscreened patients with these diagnoses.^12^ It is useful to be aware of the relation between intra-abdominal solid organ malignancy and increased risk of MVT. Thus, intra-abdominal cancer should be excluded in patients with MVT.^12^ Although there was no difference in medication with anticoagulation between patients with MVT and no AMI, it is of great concern that 28% of patients with MVT were on anticoagulation therapy at onset of disease. The history of anticoagulation therapy in patients with MVT was seldom reported in a recent systematic review of eleven contemporary retrospective studies,^2^ and the anticoagulation failure rate was found to be 1.7% in one of those studies.^12^ However, the high anticoagulation therapy failure rate presented in this prospective international study is more generalizable in different settings. The reasons behind this real-life scenario can only be speculated upon, but it is not unlikely that a large proportion of these MVT patients on anticoagulation have no or poor adherence to this preventive medication as adherence to medication in chronic diseases commonly is reported to be around 50%.^13^

There is an urgent need to find an accurate plasma biomarker for all subtypes of AMI.^14^ Even though D-dimer performed better than the inflammatory marker CRP in diagnosing MVT in the adjusted analysis, D-dimer does not seem to be sufficiently predictive to be of diagnostic aid based on the results of this matched case-control study. Even if 100% of patients with MVT had values above the upper reference value, 79% of patients had elevated levels also in the control group. Thus, normal D-dimer might act as a test of exclusion for MVT in the present study, whereas the lack of specificity greatly reduces the D-dimer test’s utility as a useful diagnostic modality. Appropriately, a prospective multi-centre study on biomarkers in prediction of acute mesenteric ischaemia (BIPAMI study) with collection of sequential blood samples after onset of suspected AMI is in progress (ClinicalTrials.gov: NCT06212921).

The AMESI Study was not designed primarily as a laboratory study, and consequently, there existed various commercially available D-dimer assays, calibrators, and monoclonal antibodies, provided by different manufacturers^15^ (Table 1), and the results had to be pooled. Hence, the results should be interpreted cautiously due to inconsistencies in performance characteristics of the different D-Dimer assays. However, the use of automated immunoassays, of which the majority used immunoturbidimetry assay technology, with rapid sample turnaround time at all sites which provided information on the D-dimer assays, was favoured compared to more manual methods such as immunofiltration or enzyme-linked immunosorbent assay (ELISA). Analysis of plasma D-dimer in one core lab using one assay technology and calibrator, would, nevertheless introduce scientific rigour and provide more high-quality data.

An interesting observation was that plasma lactate levels were lower at measurement points 12 to 24 hours and 0 to 12 hours before diagnosis of MVT compared to non-AMI patients (Figure 2). In addition, there was a significant increase in plasma lactate levels between these 2 time points in the non-AMI group compared to the MVT group. Hence, monitoring dynamics of plasma lactate appears to be of little value in diagnosing MVT early. MVT may be secondary to acute pancreatitis in a minor proportion of patients.^16^ The finding of lower amylase levels in the MVT group may, nevertheless, be related to lower frequency of acute pancreatitis, compared to the non-AMI group. In contrast to AMI of arterial origin,^17^ it is unlikely that AMI of venous origin may lead to significant ischaemic injury to pancreas with elevation of amylase. Troponin T is a marker of myocardial stress and was reported to be elevated in 42% of patients with acute abdomen.^18^ The lower troponin T levels in MVT patients, compared to non-AMI patients, may reflect less cardiomyocyte injury due to lower age, low proportion of atherosclerotic disease, and better renal function.^17^

Even though CT of abdomen with intravenous contrast enhancement has developed as the diagnostic modality of choice for MVT,^19^ there are concerns of overuse of emergency CT scans in some healthcare systems^20^ and of its great cost.^21^ In addition, a large proportion of patients in different settings around the world must pay for diagnostic workup and treatment, and many patients cannot themselves afford expensive investigations. Therefore, laboratory tests that accurately indicate either acute vessel occlusion or intestinal ischaemia, and subsequent need of emergency CT scan, are highly warranted.

There are additional limitations of the study besides the variability of D-dimer assays. One is the unknown exact timing of blood sampling in relation to symptom duration, influencing levels of plasma biomarkers. The laboratory data was not prespecified in the AMESI Study, and this post-hoc analysis study was based on available data. Even though the number of patients with MVT tested for D-Dimer in each group was larger than ever previously reported, the sample sizes in each group, 23 in the MVT group and 34 patients in the non-AMI group, respectively, are still low, with risk of type II statistical error. Testing of D-dimer was higher in the MVT group, which may be influenced by testing of D-dimer immediately after a diagnostic CT of the abdomen in some patients. Complete data on final diagnosis was not available in the non-AMI group. The major strengths are related to the prospective case-control study design on patients with AMI, a notably very difficult-to-study group of patients, the control group consisting of patients with suspected but confirmed non-AMI patients, and involvement of study sites in various countries increasing generalizability of the findings.

In conclusion, elevated D-dimer was associated with MVT, but the specificity of D-dimer was, however, poor. Currently, diagnosis of MVT is almost exclusively dependent on CT of the abdomen with intravenous contrast enhancement and imaging in the portal phase. There is an urgent need to find a more accurate plasma biomarker for this condition.

## Supplemental Material

sj-docx-1-bmi-10.1177_11772719241296631 – Supplemental material for D-Dimer in Acute Mesenteric Venous Thrombosis: A Prospective Case-Control International Multicenter Study Supplemental material, sj-docx-1-bmi-10.1177_11772719241296631 for D-Dimer in Acute Mesenteric Venous Thrombosis: A Prospective Case-Control International Multicenter Study by Stefan Acosta, Annika Reintam Blaser, Alexandre Nuzzo, Yasmin Soltanzadeh-Naderi, Joel Starkopf, Alastair Forbes, Marko Murruste, Kadri Tamme, Anna-Liisa Voomets, Merli Koitmäe, Miklosh Bala, Zsolt Bodnar, Dumitru Casian, Zaza Demetrashvili, Alan Biloslavo, Virginia Dúran Muñoz-Cruzado, Benjamin Hess, Karri Kase, Mikhail Kirov, Matthias Lindner, Cecilia I Loudet, Dimitrios Damaskos and Martin Björck in Biomarker Insights

## Declarations

**Ethics approval and consent to participate:** Primary ethical approval was obtained from the Ethics Committee of the University of Tartu (357/T-8 and 364M-7). Each participating site obtained local Ethics Committee approval according to site, country, and institutional regulations. Delayed written informed consent was obtained from the patient or patient’s next of kin/proxy at the first possibility if requested by the local ethics committee.

**Consent for publication:** Written informed consent for publication was provided by the participant(s) or a legally authorized representative.

**Competing interests:** The author(s) declared the following potential conflicts of interest with respect to the research, authorship, and/or publication of this article: ARB has received speaker or consultancy fees from Nestlé, VIPUN Medical, Nutricia and Fresenius Kabi, and is holding a grant from Estonian Research Council (PRG1255). AF has received speaker fees from B Braun and Fresenius Kabi. AN has received speaker or consultancy fees from Abbvie and Janssen, research funding from MSD-Avenir, and PhD grants from Fondation de l’Avenir and SNFGE. SA, YSN, JS, MM, KT, ALV, MK, MBa, ZB, DC, ZD, AB, VDMC, BH, KK, MK, ML, CIL, DD and MBj declare no conflicts of interest.

**Availability of data and materials:** The raw data supporting the conclusions of this article will be made available on request by the authors.
