# Supplementary material for: D-Dimer in Acute Mesenteric Venous Thrombosis: A Prospective Case-Control International Multicenter Study
Source: Biomark Insights. 2024 Nov 26;19:11772719241296631. doi: 10.1177/11772719241296631 (PMC11590162; doi:10.1177/11772719241296631)
Supplement: sj-docx-1-bmi-10.1177_11772719241296631 – Supplemental material for D-Dimer in Acute Mesenteric Venous Thrombosis: A Prospective Case-Control International Multicenter Study [file sj-docx-1-bmi-10.1177_11772719241296631.docx]

**Supplementary Table S1.** STROBE Statement—checklist of items that should be included in reports of observational studies

|  | |  | Item No. | Recommendation | Page  No. | Relevant text from manuscript |
| --- | --- | --- | --- | --- | --- | --- |
| **Title and abstract** | |  | 1 | (*a*) Indicate the study’s design with a commonly used term in the title or the abstract | 1 | The title. |
|  |  |  |  | (*b*) Provide in the abstract an informative and balanced summary of what was done and what was found | 7 | The abstract. |
|  | Introduction | | | | |  |
| Background/rationale | |  | 2 | Explain the scientific background and rationale for the investigation being reported | 8 | Introduction, paragraph 1 and 2 |
| Objectives | |  | 3 | State specific objectives, including any prespecified hypotheses | 8 | Introduction, last paragraph (3) |
|  | Methods | | | | |  |
| Study design | |  | 4 | Present key elements of study design early in the paper | 9 | Methods: Study design |
| Setting | |  | 5 | Describe the setting, locations, and relevant dates, including periods of recruitment, exposure, follow-up, and data collection | 9 | Methods: Study sample |
| Participants | |  | 6 | (*a*) *Cohort study*—Give the eligibility criteria, and the sources and methods of selection of participants. Describe methods of follow-up  *Case-control study*—Give the eligibility criteria, and the sources and methods of case ascertainment and control selection. Give the rationale for the choice of cases and controls  *Cross-sectional study*—Give the eligibility criteria, and the sources and methods of selection of participants | 9 | Methods: Study sample and Study objectives |
|  |  |  |  | (*b*) *Cohort study*—For matched studies, give matching criteria and number of exposed and unexposed  *Case-control study*—For matched studies, give matching criteria and the number of controls per case | - | Not applicable |
| Variables | |  | 7 | Clearly define all outcomes, exposures, predictors, potential confounders, and effect modifiers. Give diagnostic criteria, if applicable | 9 | Methods: Study sample and Definitions |
| Data sources/ measurement | |  | 8* | For each variable of interest, give sources of data and details of methods of assessment (measurement). Describe comparability of assessment methods if there is more than one group | 9 | Methods: Study sample |
| Bias | |  | 9 | Describe any efforts to address potential sources of bias | 10 | Methods: Statistics |
| Study size | |  | 10 | Explain how the study size was arrived at | 9 | Methods: Study sample |

| Quantitative variables |  | 11 | Explain how quantitative variables were handled in the analyses. If applicable, describe which groupings were chosen and why | 10 | Methods: Statistics |
| --- | --- | --- | --- | --- | --- |
| Statistical methods |  |  | (*a*) Describe all statistical methods, including those used to control for confounding | 10 | Methods: Statistics |
|  |  |  | (*b*) Describe any methods used to examine subgroups and interactions | - | Not applicable |
|  |  | 12 | (*c*) Explain how missing data were addressed | 10 | Methods: Statistics. No imputation of missing data |
|  |  |  | (*d*) *Cohort study*—If applicable, explain how loss to follow-up was addressed  *Case-control study*—If applicable, explain how matching of cases and controls was addressed  *Cross-sectional study*—If applicable, describe analytical methods taking account of sampling strategy | 10 | Methods: Statistics |
|  |  |  | (*e*) Describe any sensitivity analyses | - | Not applicable |
| Results | | | | | |
| Participants |  | 13* | (a) Report numbers of individuals at each stage of study—eg numbers potentially eligible, examined for eligibility, confirmed eligible, included in the study, completing follow-up, and analysed | 11 | Results: Comparing of clinical background between patients with acute MVT and control group (Table 1) |
|  |  |  | (b) Give reasons for non-participation at each stage | - | Not applicable |
|  |  |  | (c) Consider use of a flow diagram | - | Not applicable. |
| Descriptive data |  | 14** | (a) Give characteristics of study participants (eg demographic, clinical, social) and information on exposures and potential confounders | 11 | Results: Comparing of clinical background (Table 1), clinical (Table 2) and laboratory (Table 3) data at admission between patients with acute MVT and control group |
|  |  |  | (b) Indicate number of participants with missing data for each variable of interest | 16-18 | Tables 1 - 3 |
|  |  |  | (c) *Cohort study*—Summarise follow-up time (eg, average and total amount) | - | Not applicable |
| Outcome data |  | 15* | *Cohort study*—Report numbers of outcome events or summary measures over time | - | Not applicable |
|  |  |  | *Case-control study—*Report numbers in each exposure category, or summary measures of exposure | - | Not applicable. Diagnostic study |
|  |  |  | *Cross-sectional study—*Report numbers of outcome events or summary measures |  | Not applicable |
| Main results |  | 16 | (*a*) Give unadjusted estimates and, if applicable, confounder-adjusted estimates and their precision (eg, 95% confidence interval). Make clear which confounders were adjusted for and why they were included | 16-19 | Tables 1 - 4 |
|  |  |  | (*b*) Report category boundaries when continuous variables were categorized | - | Not applicable. |
|  |  |  | (*c*) If relevant, consider translating estimates of relative risk into absolute risk for a meaningful time period | - | Not applicable. |
| Other analyses |  | 17 | Report other analyses done—eg analyses of subgroups and interactions, and sensitivity analyses | - | Not applicable |
| Discussion | | | | | |
| Key results |  | 18 | Summarise key results with reference to study objectives | 12 | Discussion: Paragraph 1 |
| Limitations |  | 19 | Discuss limitations of the study, taking into account sources of potential bias or imprecision. Discuss both direction and magnitude of any potential bias | 13 | Discussion: Paragraph 6 |
| Interpretation |  | 20 | Give a cautious overall interpretation of results considering objectives, limitations, multiplicity of analyses, results from similar studies, and other relevant evidence | 13-14 | Discussion: Conclusion (Paragraph 7) |
| Generalisability |  | 21 | Discuss the generalisability (external validity) of the study results | 13 | Discussion: Paragraph 6 |
| Other information | | |  | | |
| Funding |  | 22 | Give the source of funding and the role of the funders for the present study and, if applicable, for the original study on which the present article is based | 2 | Funding statement |

**Note:** An Explanation and Elaboration article discusses each checklist item and gives methodological background and published examples of transparent reporting. The STROBE checklist is best used in conjunction with this article (freely available on the Web sites of PLoS Medicine at http://www.plosmedicine.org/, Annals of Internal Medicine at http://www.annals.org/, and Epidemiology at http://www.epidem.com/). Information on the STROBE Initiative is available at www.strobe-statement.org
